# Multi-step genomic dissection of a suspected intra-hospital *Helicobacter cinaedi* outbreak

**DOI:** 10.1099/mgen.0.000236

**Published:** 2019-01-17

**Authors:** Yasuhiro Gotoh, Takako Taniguchi, Dai Yoshimura, Keisuke Katsura, Yuji Saeki, Yasutoshi Hirabara, Mayumi Fukuda, Ichiro Takajo, Junko Tomida, Yoshiaki Kawamura, Yoshitoshi Ogura, Takehiko Itoh, Naoaki Misawa, Akihiko Okayama, Tetsuya Hayashi

**Affiliations:** ^1^​ Department of Bacteriology, Faculty of Medical Sciences, Kyushu University, Fukuoka, Japan; ^2^​ Previous address: Division of Microbiology, Department of Infectious Diseases, Faculty of Medicine, University of Miyazaki, Miyazaki, Japan; ^3^​ Laboratory of Veterinary Public Health, Department of Veterinary Medical Science, Faculty of Agriculture, University of Miyazaki, Miyazaki, Japan; ^4^​ Graduate School of Bioscience and Biotechnology, Tokyo Institute of Technology, Tokyo, Japan; ^5^​ Frontier Science Research Center, University of Miyazaki, Miyazaki, Japan; ^6^​ Center for Infection Control, University of Miyazaki Hospital, Miyazaki, Japan; ^7^​ Department of Microbiology, School of Pharmacy, Aichi Gakuin University, Nagoya, Japan; ^8^​ Faculty of Agriculture, University of Miyazaki Center for Animal Disease Control, University of Miyazaki, Miyazaki, Japan; ^9^​ Department of Rheumatology, Infectious Diseases and Laboratory Medicine, University of Miyazaki, Miyazaki, Japan

**Keywords:** intra-hospital outbreak, *Helicobacter cinaedi*, asymptomatic carrier, antimicrobial resistance, multi-step phylogenetic analysis

## Abstract

*Helicobacter cinaedi* is an emerging pathogen causing bacteraemia and cellulitis. Nosocomial transmission of this microbe has been described, but detailed molecular-epidemiological analyses have not been performed. Here, we describe the results of a multi-step genome-wide phylogenetic analysis of a suspected intra-hospital outbreak of *H. cinaedi* that occurred in a hospital in Japan. The outbreak was recognized by the infectious control team (ICT) of the hospital as a sudden increase in *H. cinaedi* bacteraemia. ICT defined this outbreak case based on 16S rRNA sequence data and epidemiological information, but were unable to determine the source and route of the infections. We therefore re-investigated this case using whole-genome sequencing (WGS). We first performed a species-wide analysis using publicly available genome sequences to understand the level of genomic diversity of this under-studied species. The clusters identified were then separately analysed using the genome sequence of a representative strain in each cluster as a reference. These analyses provided a high-level phylogenetic resolution of each cluster, identified a confident set of outbreak isolates, and discriminated them from other closely related but distinct clones, which were locally circulating and invaded the hospital during the same period. By considering the epidemiological data, possible strain transmission chains were inferred, which highlighted the role of asymptomatic carriers or environmental contamination. The emergence of a subclone with increased resistance to fluoroquinolones in the outbreak was also recognized. Our results demonstrate the impact of the use of a closely related genome as a reference to maximize the power of WGS.

## Data Summary

The raw read sequences and assembled scaffold sequences obtained in this study have been deposited in GenBank/EMBL/DDBJ under the BioProject accession number PRJDB6556.

**Impact Statement**

Whole-genome sequencing (WGS) can distinguish closely related bacterial strains isolated in outbreaks. Many WGS analyses of intra-hospital outbreaks have been reported, primarily for well-known pathogens whose genomic information has been well accumulated. However, this approach can theoretically be applied to any pathogen, even if only a limited amount of genomic information is available for the suspected causative agent, such as *Helicobacter cinaedi*, for which only four genome sequences are currently available. Here, we present the results of a multi-step WGS analysis of a suspected intra-hospital outbreak of *H. cinaedi*; this analysis comprised (i) a species-wide analysis using a publicly available genome sequence as a reference to understand the level of genetic relatedness of the test strains, and (ii) analyses of individual clusters identified in the first step using a representative genome in each cluster as a reference. This approach confidently identified outbreak strains and, by considering the epidemiological data, revealed complicated strain transmission routes. Our results demonstrate the impact of the use of a closely related genome as a reference to maximize the power of WGS. As appropriate reference sequences are not always available for minor or under-studied pathogens, our approach would be a powerful strategy applicable to any pathogen.

## Introduction

*Helicobacter cinaedi*, a member of the enterohepatic *Helicobacter* colonizing intestinal tracts, is an emerging opportunistic pathogen, while *Helicobacter pylori*, the well-known and best-studied species of genus *Helicobacter*, is classified as gastric *Helicobacter* [1, 2] (see the 16S RNA-based phylogenetic tree reported in our previous paper [2] for the phylogenetic position of this species in genus *Helicobacter*). Although *H. cinaedi* was first isolated from an immunocompromised patient [3], it has also been isolated from immune-competent patients and healthy persons [4–7], and causes bacteraemia, cellulitis and other conditions such as arthritis and meningitis [8–11]. In many reported cases, strains were isolated from blood, but the bacteria have also been detected by PCR or isolated from faecal samples from patients and healthy volunteers [12–14], suggesting that bacterial translocation from the gastrointestinal tract may be a route of infection, although the details have yet to be elucidated [2, 15]. The global frequency of *H. cinaedi* infections is also very poorly understood, and most studies on this species have been conducted in Japan. In these reports, the positive rates of *H. cinaedi* among positive blood cultures ranged from 0.22 % [16] to 2.2 % [15]. The estimated mortality rate of *H. cinaedi* bacteraemia is 6.3 % [15]. Although the prognosis is generally good, recurrence of bacteraemia is frequently observed, with reported rates of 30–60 % [2] or 72.7 % [17]. Although several reports recommended combination chemotherapy with an extended duration [4, 18, 19], no guideline for the usage of antimicrobials has been established. Antimicrobial-resistance genes encoded by mobile genetic elements such as plasmids and transposons have not yet been identified in *H. cinaedi*. Several intra-hospital outbreaks of *H. cinaedi* have been reported, but detailed molecular-epidemiological analyses have not yet been conducted [5, 20–22].

Molecular-epidemiological analyses are required to identify the routes and/or sources of transmission of nosocomial infections. Traditional methods of detecting nosocomial transmission, such as pulse-field gel electrophoresis, are available for *H. cinaedi* [5, 20–22], but these methods do not provide sufficient resolution to distinguish closely related isolates. Molecular-epidemiological analyses using whole-genome sequencing (WGS) could, however, provide this level of resolution. Many studies using WGS have been performed, primarily for bacteria that are well known to cause nosocomial infections and whose genomic information has been well accumulated; these bacteria include meticillin-resistant *Staphylococcus aureus* [23, 24], *Klebsiella pneumoniae* [25], *Clostridium difficile* [26] and *Enterococcus faecium* [27]. However, this approach can theoretically be applied to any pathogen, even if only a limited amount of genomic information is available for the suspected causative agent, as recently shown for *Elizabethkingia anopheles* [28]. *H. cinaedi* is also one of such pathogens, for which only four genome sequences are currently available [29–31].

Here, we describe the results of a multi-step genome-wide phylogenetic analysis of a suspected intra-hospital outbreak of *H. cinaedi*. We first performed a species-wide analysis using a publicly available complete genome sequence. Clusters identified were then separately analysed using the genome sequences of strains included in each cluster as references. This series of analyses notably increased the level of resolution in each cluster. By combining the results with an investigation of clinical records for each patient, we identified an intra-hospital outbreak of a *H. cinaedi* clone and discriminated these infections from infections with other closely related but distinct clones that occurred during the same period. The identified possible strain transmission chains highlighted the role of asymptomatic carriers (ACs). The emergence of a clone with increased resistance to fluoroquinolones (FQs) in the outbreak is also described.

## Methods

### Microbiological analyses

We analysed 22 *H. cinaedi* clinical isolates obtained from 21 bacteraemia patients (P01–P21) hospitalized in hospital A in Miyazaki, Japan. All *H. cinaedi* samples were isolated from blood cultures obtained as part of routine clinical investigation using BACTEC FX (Nippon Becton Dickinson). Cultures from patients P11 and P17 were obtained when they visited the hospital after discharge. Gram-negative bacteria with a spiral form grown in blood cultures were inoculated on trypticase soy agar II containing 5 % sheep blood (Nippon Becton Dickinson) and cultivated under microaerophilic conditions (8 % CO_2_) for 3 days at 37 °C. Species were identified based on their morphological characteristics and 16S rRNA sequences. Strains CCUG18818^T^, CCUG19503 and CCUG19504 were obtained from the culture collection at the University of Göteborg (Sweden). Strains PAGU617, PAGU628 and PAGU1382 are clinical strains isolated in regions other than Miyazaki in Japan [32]. Strains analysed in this study are listed in Table 1. Antimicrobial-susceptibility testing was conducted using the agar dilution method [33].

**Table 1.** Strains used in this study n/a, Not available.

Strain	Place of strain isolation	Conclusion of ICT investigation	Patient	Ward	Time from P01D0000 (days)	Genome sequencing	Assembly status	Reference
P01D0000	Miyazaki, Japan	Not investigated	P01	Fa	0	This study	Complete	This study
P02D0213	Miyazaki, Japan	Not investigated	P02	Fa	213	This study	Draft	This study
P03D0629	Miyazaki, Japan	Outbreak	P03	Fa	629	This study	Complete	This study
P04D0736	Miyazaki, Japan	Outbreak	P04	Fa	736	This study	Draft	This study
P05D0741	Miyazaki, Japan	Outbreak	P05	Fa	741	This study	Draft	This study
P06D0798	Miyazaki, Japan	Non-outbreak	P06	Fa	798	This study	Complete	This study
P07D0876	Miyazaki, Japan	Outbreak	P07	Fa	876	This study	Draft	This study
P08D0905	Miyazaki, Japan	Outbreak	P08	Fa	905	This study	Draft	This study
P09D0927	Miyazaki, Japan	Outbreak	P09	Fa	927	This study	Draft	This study
P10D0937	Miyazaki, Japan	Outbreak	P10	Fa	937	This study	Draft	This study
P11D0946	Miyazaki, Japan	Outbreak	P11	Fa	946	This study	Draft	This study
P11D1015	Miyazaki, Japan	Outbreak	P11	Fa	1015	This study	Draft	This study
P12D0946	Miyazaki, Japan	Outbreak	P12	Fb	946	This study	Draft	This study
P13D0979	Miyazaki, Japan	Outbreak	P13	Fa	979	This study	Draft	This study
P14D1067	Miyazaki, Japan	Outbreak	P14	Fa	1067	This study	Draft	This study
P15D1072	Miyazaki, Japan	Outbreak	P15	Fc	1072	This study	Draft	This study
P16D1106	Miyazaki, Japan	Outbreak	P16	Fa	1106	This study	Draft	This study
P17D1144	Miyazaki, Japan	Outbreak	P17	Fa	1144	This study	Draft	This study
P18D1268	Miyazaki, Japan	Non-outbreak	P18	Fa	1268	This study	Draft	This study
P19D1315	Miyazaki, Japan	Non-outbreak	P19	Fa	1315	This study	Draft	This study
P20D1835	Miyazaki, Japan	Not investigated	P20	Fa	1835	This study	Draft	This study
P21D1863	Miyazaki, Japan	Not investigated	P21	Fd	1863	This study	Draft	This study
ATCC BAA-847^T^	USA	n/a	n/a	n/a	n/a	AP012492.1	Complete	[30]
CCUG18818^T^	USA	n/a	n/a	n/a	n/a	This study	Draft	[48]
CCUG19503	Ottawa, Canada	n/a	n/a	n/a	n/a	This study	Draft	[49]
CCUG19504	Ottawa, Canada	n/a	n/a	n/a	n/a	This study	Draft	[49]
MRY08-1234	Hokkaido, Japan	n/a	n/a	n/a	n/a	AP017374.1	Complete	[31]
MRY12-0051	Hokkaido, Japan	n/a	n/a	n/a	n/a	DRR090193	Draft	[31]
PAGU611	Kumamoto, Japan	n/a	n/a	n/a	n/a	AP012344.1	Complete	[29]
PAGU617	Kumamoto, Japan	n/a	n/a	n/a	n/a	This study	Draft	[32]
PAGU628	Tokyo, Japan	n/a	n/a	n/a	n/a	This study	Draft	This study
PAGU1382	Kumamoto, Japan	n/a	n/a	n/a	n/a	This study	Draft	This study

### Genome sequencing

Strains sequenced in this study were grown on Brucella agar (Nippon Becton Dickinson) with 5 % defibrinated horse blood (Kohjin Bio) for 2 days at 37 °C. Genomic DNA was purified from cells collected from the plates using the Genomic-tip 100/G system (QIAGEN) and then used to prepare sequencing libraries with the Nextera DNA library preparation kit (Illumina). Libraries were sequenced using Illumina MiSeq to obtain 151 or 251 bp paired-end (PE) reads. Sequence assembly was performed using Platanus 1.2.2 with the parameter for bubble crush (–u 0) [34]. To obtain the complete sequences of strains P01D0000, P03D0629 and P06D0798, 8 kb mate-paired libraries were prepared with the Nextera mate pair sample prep kit (Illumina) and sequenced using MiSeq to obtain mate-pair (MP) reads. PE and MP reads for each strain were assembled together using Platanus 1.2.2 with the parameter for bubble crush (–u 0), and remaining gaps were closed by PCR amplification and capillary sequencing of PCR products.

Annotation was performed by Prokka with default parameters [35]. The complete genome sequences of strains PAGU611 [29], ATCC BAA-847^T^ [30] and MRY08-1234 [31] were downloaded from the website of the National Center for Biotechnology Information (NCBI; ftp://ftp.ncbi.nlm.nih.gov/genomes/genbank/bacteria/). The sequence reads of strain MRY12-0051 [31] were obtained from the DDBJ Sequence Read Archive (DRA). All sequences determined in this study have been deposited in GenBank/EMBL/DDBJ under the BioProject accession number PRJDB6556.

### Single-nucleotide polymorphism (SNP) detection and phylogenetic analyses

Draft (only contigs of ≥1 kb in length were used) or complete sequences were aligned to reference genome sequences using NUCmer [36] with the parameters ‘—mum’. Alignments of <99 % identity and <2 kb were filtered out from the analyses to exclude possible sequences from mobile genetic elements. Only ≥500 bp regions shared by all strains tested in each analysis were used for SNP calling. To obtain only high-confidence SNPs, SNPs were excluded if they resided (i) in repetitive regions, (ii) within 100 bases of alignment boundaries, (iii) in the immediate vicinity (within a 5 bp distance) of any insertion/deletion sites or (iv) in regions that possibly experienced homologous recombination as predicted by Gubbins with default parameters [37]. Maximum-likelihood (ML) phylogenetic trees were reconstructed using RAxML-NG (https://github.com/amkozlov/raxml-ng/) with an inferred model by ModelTest (https://github.com/ddarriba/modeltest/) and 1000 bootstrap replicates [38]. Monophyletic clades, referred to as clusters, were identified using TreeGubbins (https://github.com/simonrharris/tree_gubbins/) with default parameters. A neighbour-joining tree was inferred with the p-distance method in mega7 [39]. The temporal signal was investigated using TempEst [40].

## Results

### Cases of *H. cinaedi* infection

**Fig. 1.**
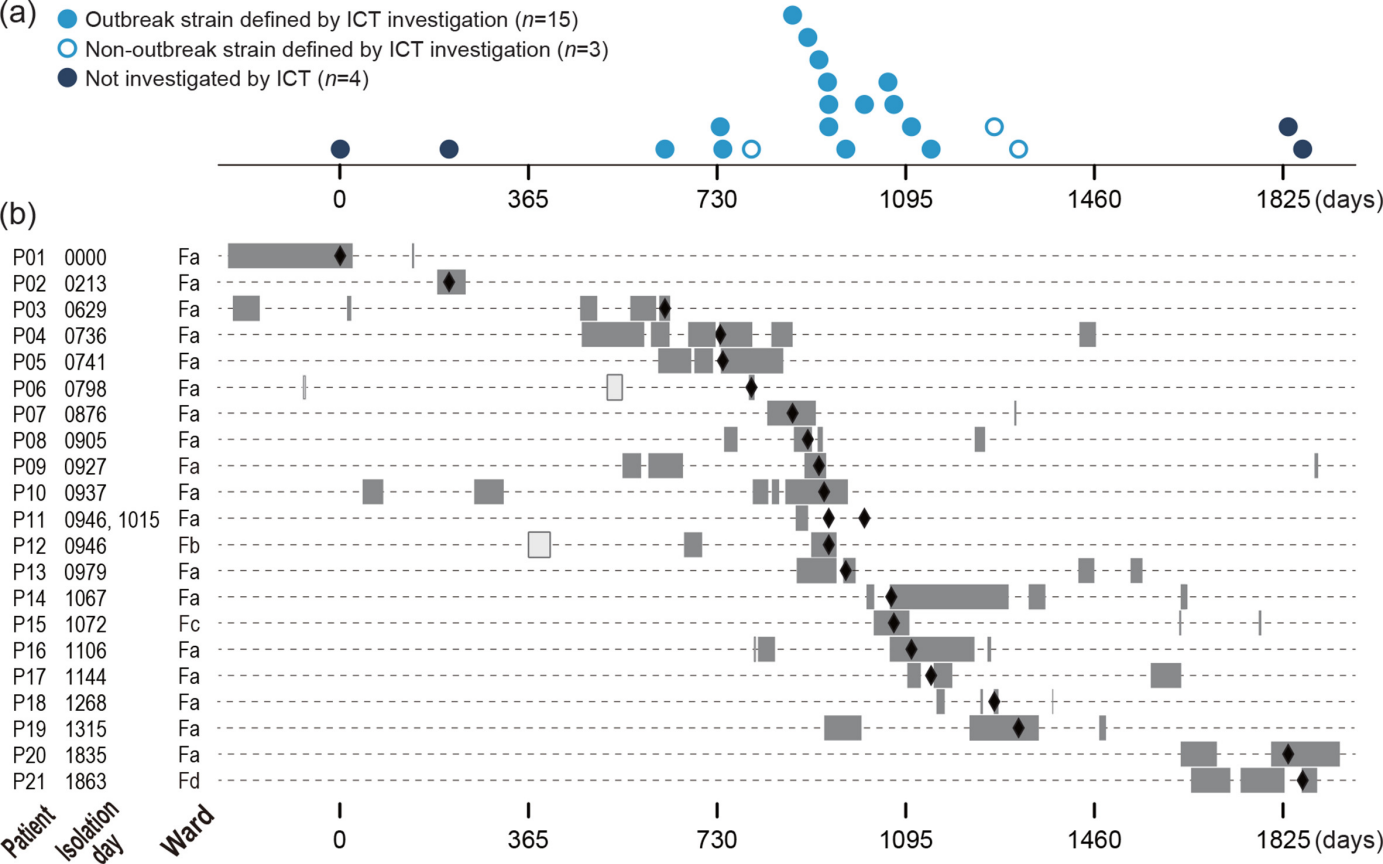
The 22 *H. cinaedi* strains isolated in hospital A in Miyazaki used for the re-investigation by WGS analysis of the suspected outbreak. (a) The time of strain isolation (the number of days after the isolation of P01D0000, the earliest isolate in the present strain set) is shown for each strain. (b) The hospitalization periods and admitted wards of the 21 patients (grey bars). Open bars in patients P06 and P12 indicate the admission to other wards. The dates of strain isolation are indicated by closed diamonds.

The infectious control team (ICT) of hospital A in Miyazaki, Japan, recognized a sudden increase in *H. cinaedi* bacteraemia cases in the hospital, with 17 cases identified during a 2 year period (Fig. 1). Prior to this period, only a few or no cases (up to two cases) were identified annually in the hospital. Thus, the team suspected the occurrence of an intra-hospital outbreak of *H. cinaedi* and investigated the genetic relationship of the isolates obtained from these patients (18 isolates, including 2 from patient P11, from whom *H. cinaedi* was isolated twice) based on their 16S rRNA sequences. By comparing the sequences and the epidemiological information, the ICT defined one outbreak case based on the following criteria: (i) isolates had the same 16S rRNA sequences and (ii) the duration of hospitalization of the patients overlapped. The defined outbreak included 14 cases/patients (15 isolates including 2 from P11; indicated by blue closed circles in Fig. 1). These patients were hospitalized in the same ward (ward Fa), except for patient P12 (ward Fb). The 16S rRNA sequences of the other three isolates (from P06, P18 and P19) were identical but different from that of the outbreak isolates. Patients P18 and P19 were both hospitalized in ward Fa during an overlapping period, suggesting strain transmission between them, but there was no overlap of the hospitalization period between P06 and P18/19.

In this investigation, it was not possible to determine the source and route of *H. cinaedi* infections in the outbreak. Therefore, we re-investigated this outbreak case by WGS. In this re-investigation, the three non-outbreak isolates mentioned above and four blood isolates, which were isolated in the hospital before and after the outbreak period (during the preceding 2 years and subsequent 2 years), were included. Thus, a total of 22 blood isolates from 21 patients, comprising 8 males and 13 females with a median age of 61 years (range 27 to 79 years), from the same hospital were analysed. As shown in Table S1 (available with the online version of this article), isolates were named using the patient ID (the prefix P followed by the patient number) and the day of isolation (the prefix D followed by the number of days after the day of the isolation of P01D0000, the earliest isolate in the present strain set). However, for convenience and simplicity, a strain isolated from patient PXX is referred to as ‘isolate PXX’ in the text below. The two isolates from patient P11 are referred to as ‘isolate P11a’ (the first isolate) and ‘isolate P11b’ (the second isolate).

### The first-step phylogenetic analysis

**Fig. 2.**
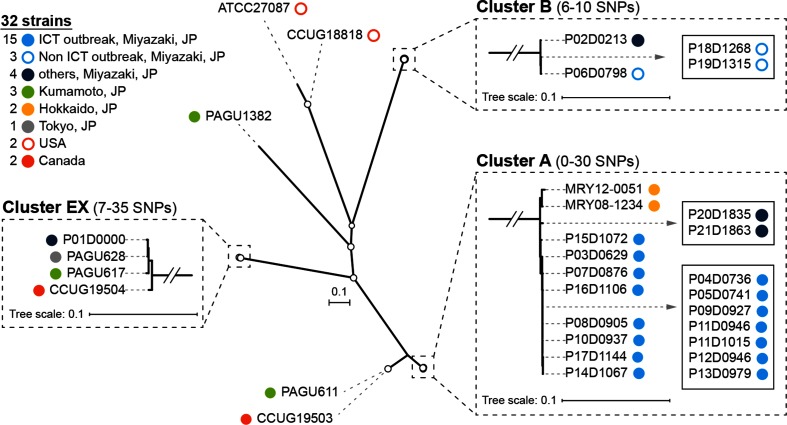
The first-step WGS-based phylogenetic analysis. A species-wide analysis of *H. cinaedi* was performed using 32 strains (22 Miyazaki strains and 10 strains from various other regions) and the complete genome sequence of strain PAGU611 as a reference. Based on the 4154 SNPs identified, an unrooted ML tree was reconstructed using RAxML-NG with the transversion model (TVM). Nodes with ≥80 % bootstrap support based on 1000 bootstrap replicates are indicated by open circles. Three monophyletic clusters identified by TreeGubbins are shown in the boxes outlined with dashed lines, and indistinguishable strains in each cluster are shown in solid boxes. Bars, number of substitutions per site. JP, Japan.

To determine the genetic relationship of the 22 *H. cinaedi* isolates, we obtained their draft sequences with total contig lengths ranging from 2.04 to 2.24 Mb and roughly comparable to previously sequenced strains (Table S1). In addition, to understand their genetic relationship in the context of the genomic diversity of this species, we obtained genome sequences of an additional 10 strains isolated in various geographical regions in and out of Japan (Tables 1 and S1; including three finished genomes) and performed a WGS-based phylogenetic analysis of the 32 *H. cinaedi* strains. We first used the PAGU611 genome as a reference. This analysis identified 4154 SNPs in 870 875 informative sites shared by the 32 strains. In an ML phylogenetic tree reconstructed based on these SNPs, the presence of three monophyletic clusters, which contained four or more strains, was evident (clusters A, B and EX; Fig. 2). SNP distances between the three clusters were 1927–1945 SNPs (between clusters A and B), 1476–1492 (clusters A and EX) and 1902–1915 (clusters B and EX). Cluster A contained two strains isolated in Hokkaido, Japan, and 17 Miyazaki isolates. These strains exhibited 0–30 SNP differences from each other. All 15 outbreak isolates defined by the ICT, together with isolates P2 and P21 isolated in the same hospital after the ICT investigation, belonged to cluster A. Cluster B contained four Miyazaki isolates with 6–10 SNP distances. Three of these were defined as non-outbreak isolates by the ICT, and one (isolate P02) was isolated before the ICT investigation. Cluster EX contained four strains, which were within 7–35 SNP distances, but were isolated in different places. Isolate P01, the earliest isolate among the 22 Miyazaki isolates, belonged to this cluster.

The results of this analysis supported the conclusion of the ICT. However, the number of informative sites available represented less than 40 % of the entire *H. cinaedi* genome (Table S2) due to the genetic distances between the test strains and the reference strain, and the presence of relatively large amounts of recombinogenic sequences (663 kb, as deduced by Gubbins). Accordingly, seven isolates and a pair of isolates were indistinguishable in cluster A. Cluster B also included a pair of indistinguishable isolates.

### The second-step phylogenetic analyses

In the second step of the analysis, we analysed clusters separately using the complete sequence of a representative isolate of each cluster as a reference to obtain higher-resolution data.

#### Cluster A

**Fig. 3.**
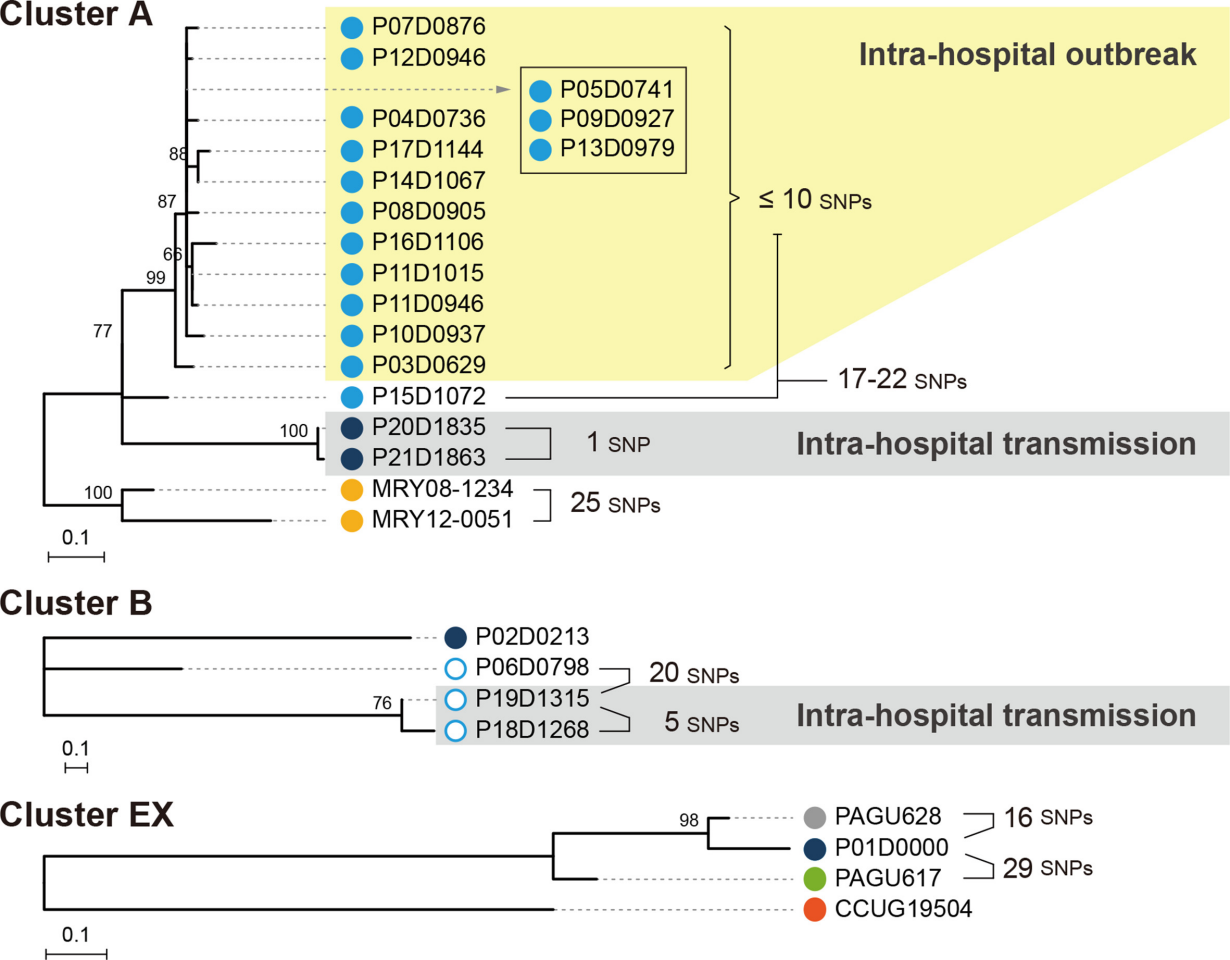
Phylogenetic analyses of the three clusters identified in the first-step analysis. The three clusters identified in the first-step analysis were separately analysed. SNPs were identified in each cluster using the complete genome sequences of MRY08-1234 (cluster A), P06D0798 (cluster B) and P01D0000 (cluster EX) as references for each cluster to reconstruct ML trees using RAxML-NG with the TPM2 (cluster A) or K80 (clusters B and EX) models. The trees are shown by mid-point rooting. Bootstrap values based on 1000 bootstrap replicates are indicated at nodes. Pairwise SNP distances between isolates are also indicated. The colours of each dot correspond to the strain information indicated in Fig. 2.

MRY08-1234 was used as a reference in this analysis. The number of available informative sites increased notably, and the number of confident SNPs identified in cluster A increased from 52 to 101 (Table S2). An ML phylogenetic tree reflecting more accurate genetic distances was reconstructed based on the 101 identified SNPs (Fig. 3). Due to this increased resolution, four of the seven isolates and a pair of isolates that were indistinguishable in the first-step analysis were distinguished.

#### Cluster B

We determined the complete genome sequence of isolate P06 and used it as a reference. The number of informative sites also increased notably, and the number of SNPs increased from 12 to 40 (Table S2). An ML tree reconstructed using the 40 SNPs (Fig. 3) distinguished the two isolates that were indistinguishable in the first-step analysis.

#### Cluster EX

The complete genome sequence of isolate P01 was determined and used as a reference. The number of informative sites also increased notably, and the number of SNPs increased from 45 to 110 (Table S2). An ML tree reconstructed using the 110 SNPs (Fig. 3) revealed that isolate P01, isolated in Miyazaki, still exhibited only a 16 SNP distance to PAGU628, isolated in Tokyo, Japan.

The result of cluster EX analysis indicated that *H. cinaedi* strains without any epidemiological links could be within a 16 SNP distance. Based on this finding, we reanalysed the data for cluster A. This analysis revealed that, among the 15 isolates defined as outbreak strains by the ICT, 14 were within a 10 SNP distance (Fig. 3; shaded yellow). Each isolate in this subset differed from the closest isolate by 0–5 SNPs. However, one of the 15 isolates (isolate P15) differed from the other 14 isolates by 17–22 SNPs. These results indicate that the 14 isolates can be regarded as true outbreak strains, while isolate P15 cannot. The results of root-to-tip analyses using TempEst [40] supported this conclusion (Table S3). The estimated time to the most recent common ancestor (TMRCA) of the 15 isolates was 3594 days before the isolation date of isolate P03 (the first isolate among the 15 isolates), while that of the 14 isolates was 372 days before the isolation of isolate P03. A pair of cluster A strains, isolates P20 and P21, also differed by only one SNP (Fig. 3; shaded by grey). A similar re-inspection of the data for cluster B revealed that isolates P18 and P19 differed by five SNPs (Fig. 3).

### The third-step analysis of the 14 outbreak strains

Among the 14 isolates we confidently defined as outbreak strains, we  were still unable to distinguish three (isolates P05, P09 and P13) in the second-step analysis (Fig. 3). As an attempt to gain further information on the genetic relationship of these three isolates, we determined the complete genome sequence of isolate P03, which was the earliest isolate among the 14 isolates, and we performed a phylogenetic analysis of the 14 strains using it as a reference. This analysis slightly increased the number of informative sites (74 kb). However, no additional SNPs were identified among the three isolates (Table S2).

### Combined analysis of genomic and epidemiological data

**Fig. 4.**
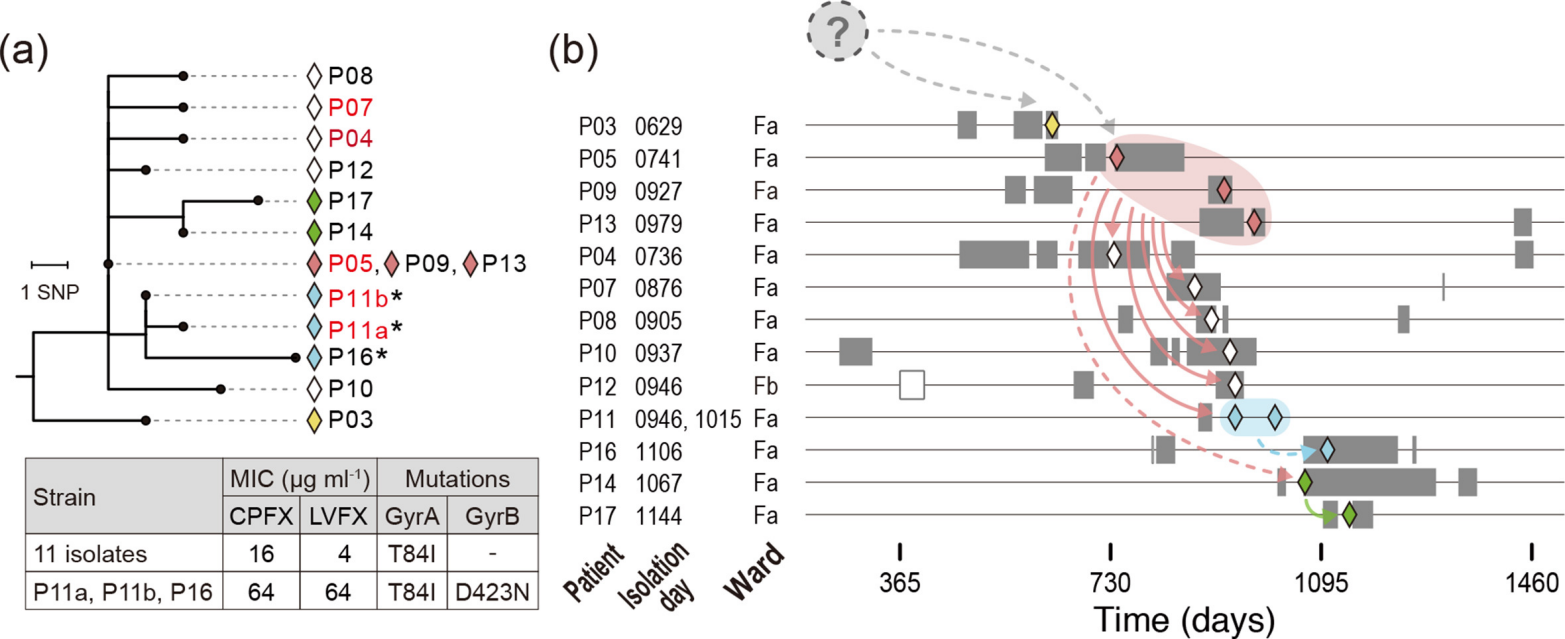
The genetic relationship of the 14 outbreak isolates and their possible transmission routes. (a) The phylogenetic relationships of the 14 outbreak isolates defined in this study are shown as a neighbour-joining tree. The tree was rooted with isolate P15. Diamonds are coloured corresponding to the inferred transmission routes shown in (b). Three isolates that acquired a higher FQ resistance are indicated by asterisks. Five patients with confirmed histories of FQ administration (within 6 months before strain isolation) are indicated in red. The MICs of the 14 isolates to ciprofloxacin (CPFX) and levofloxacin (LVFX) and the mutations (amino acid substitutions) found in GyrA and GyrB in the 14 isolates are also shown. (b) Hospitalization periods of each patient (grey bars), dates of strain isolation (coloured diamonds) and possible strain transmission routes inferred from the phylogenetic relationships of the outbreak isolates are shown. An open bar for patient P12 indicates the admission to a different ward. Dashed arrows indicate the transmissions between patients with no hospitalization period overlap, for which the involvement of ACs or environmental contamination is inferred. Note that a possibility that isolates within a one or two SNP difference (P05/P09/P13, P04, P07, P08, P11, P12 and P14, or some of them) were transmitted from a common source cannot be ruled out (see the main text for more details).

To analyse the route of strain transmission during the outbreak, we performed a combined analysis of the genomic and epidemiological data of the 14 outbreak isolates. By mapping the genetic relationships of the isolates to the hospitalization histories of patients, we identified complicated routes of strain transmission (Fig. 4).

Although isolate P03 was the first isolate among the 14 outbreak isolates, this isolate does not appear to be the ancestor of other isolates; isolate P03 was first separated from other outbreak isolates and showed five or more SNP differences to other isolates (Figs 3 and 4). The phylogenetic relationship of the remaining 13 isolates suggests that the 3 indistinguishable isolates obtained from patients P05, P09 and P13 represent the ancestor of the others. Considering the phylogenetic relationship of the 13 isolates, the clone was likely transmitted from either P05/P09/P13 to six patients (P04, P07, P08, P10, P11 and P12) and further from P11 and P14 to P16 and P17, respectively. During these transmissions, the clone spread to ward Fb (patient P12). Notably, no hospitalization period overlap was observed between P05/P09/P13 and P14 or between P11 and P16, suggesting the involvement of ACs (patients or staff) or environmental contamination in these transmission events.

The above-mentioned transmission network was deduced mainly based on the simplest interpretation of the phylogenetic relationship of the outbreak isolates. However, some caution is required for interpreting the data, because analysis of two isolates obtained from P11 suggested the presence of some level of within-host genomic diversity of *H. cinaedi* strains. The two isolates were obtained at an interval of 69 days and differed by only one SNP; however, their phylogenetic relationship indicated that the earlier isolate (P11a) was derived from the latter isolate (P11b). This suggests that a certain level of genomic diversity was already present in the *H. cinaedi* population that colonized P11, the two isolates were present within this within-host diverse population, and the duration of *H. cinaedi* colonization in P11 might have been long enough to generate this diversity. Given the presence of such within-host diversity in *H. cinaedi* populations colonized in each patient, some of the very small number SNP differences observed in this analysis might have been generated by acquiring diverse isolates from a common source (transmission of diversity). Because such SNP cannot be discriminated from those generated *de novo* after transmission, we cannot rule out a possibility that isolates within a one or two SNP difference were transmitted from a common source (P05/P09/P13, P04, P07, P08, P11, P12 and P14, or some of them).

A similar investigation of two pairs of very closely related isolates in cluster A (isolates P20 and P21; one SNP difference) and cluster B (isolates P18 and P19; five SNP differences) revealed an overlap of the hospitalization period between the two patients in each case (Fig. S1). This finding strongly suggests the occurrence of intra-hospital strain transmissions between the patients, although, in the case of cluster A isolates, the two patients were hospitalized in different wards.

### Alterations in antimicrobial resistance during the outbreak

The present study provided an opportunity to analyse the alterations of the outbreak clone (14 outbreak isolates) in antimicrobial resistance during a 17.2 month period. Determination of the minimum inhibitory concentrations (MICs) of nine antimicrobials for each isolate revealed that all 14 isolates were resistant to macrolides (Table S4). The resistance was due to a mutation in the 23S rRNA gene [22]. We confirmed that both copies of the 23S rRNA gene sequences encoded in the complete genome sequence of isolate P03 had the mutation. A more notable finding was the variation in the resistance to FQs. All 14 isolates were resistant to FQs, with MICs of ≥16 µg ml^−1^ to ciprofloxacin and ≥4 µg ml^−1^ to levofloxacin (Fig. 4, Table S4), but three isolates (isolates P11a, P11b and P16) showed a remarkable increase in FQ resistance (MICs of both FQs: 64 µg ml^−1^). All 14 isolates contained the T84I mutation in GyrA, which accounts for their basic FQ resistance [21]. In addition, the three highly resistant isolates contained the D423N mutation in GyrB, which is known to confer FQ resistance [41]. Among the patients involved in the outbreak, FQs were administered to four patients. One of them was patient P11, from whom highly resistant isolates were obtained. This finding indicates that the FQ-highly-resistant subclone emerged within patient P11 and was selected by FQ administration therein, and was then transmitted to P16, who had no history of FQ administration (Fig. 4).

## Discussion

In this study, we re-examined an intra-hospital *H. cinaedi* outbreak, which was defined by the ICT based on the 16S rRNA sequences of isolates and the patients’ hospitalization records, by multi-step WGS-based phylogenetic analyses. In the first step, a publicly available complete genome sequence with an unknown genetic relatedness to the test strains was used as a reference. Additional *H. cinaedi* strains isolated in the same hospital and in various regions inside and outside of Japan were also included to understand the level of genetic relatedness of the outbreak isolates in the species-wide context. In the second step, each cluster identified was separately analysed using the genome sequences of the representative isolates of each cluster as references. This approach provided a high level of resolution to discriminate each outbreak isolate differing by only one or a few SNPs at the WGS level (Fig. 2, Table S1). The third step of the analysis targeting only outbreak isolates did not provide a further increase in resolution, but based on the results of this series of analyses, we identified true outbreak strains with a high confidence and their precise genetic relationships. Although WGS has a high phylogenetic resolution power, our data demonstrate the impact of the use of a closely related genome as the reference to maximize its power. Appropriate reference sequences are not always available, particularly for minor or under-studied pathogens. Thus, the multi-step analysis described here is a powerful strategy applicable to any pathogen. The results of this study also highlighted the importance of understanding the potential level of genomic diversity in the species to be analysed. Our results indicated that *H. cinaedi* strains with no epidemiological link could be within a 16 SNP distance (Fig. 2). This information is probably essential to precisely and confidently defining outbreak strains in any WGS-based phylogenetic analysis.

Determining the precise genetic relationship between the outbreak isolates as well as strains isolated before and after the outbreak period revealed complicated transmission routes of the outbreak clone and a complex population structure of the *H. cinaedi* strains obtained during the study period. However, several clinically important issues to be clarified in future studies have been noted. First, understanding the genomic diversity *H. cinaedi* within each patient is essential to precisely determine the route/source of strain transmission. Due to the lack of this information (except for patient P11), one of the limitations of this retrospective study, we were unable to pinpoint the route of strain transmission in this outbreak case as described in Results. Analysis of multiple colonies from each clinical sample will further enhance the power of WGS, as reported for several pathogens [27, 42–44].

The surveillance of ACs and environmental contamination is also important to fully understand the route/source of strain transmission. In this study, the involvement of ACs (patients or staff) or environmental contamination in strain transmission was clearly suggested on multiple occasions. However, the lack of surveillance of these potential routes/sources of strain transmission, another limitation of this study, hampered further analyses of this issue. As the intestinal colonization rate of *H. cinaedi* in patients with *H. cinaedi* bacteraemia has been reported to be greater than 50 % [15] and the carriage of *H. cinaedi* has also been reported in 10 % of healthy volunteers [14], significant numbers of inpatients may have been colonized by *H. cinaedi* during the study period. Although it is not easy to isolate *H. cinaedi* from stool samples because of the lack of efficient selective media, WGS analysis of multiple isolates from each stool sample and/or isolates from longitudinal sampling should also be conducted if possible, as reported for other pathogens that asymptomatically colonize the gut, such as *Enterococcus faecium*[27], extended-spectrum-β-lactamase-producing *Escherichia coli* [44], *K. pneumoniae* [45] and *C. difficile* [46]. Such analyses would provide more information on the within-host diversity and evolution of this species and on the duration of the AC state, which are required for precisely interpreting the data from outbreak investigations using WGS.

Regarding the possible involvement of environmental contamination in strain transmission in this outbreak, it may be noteworthy that, after the ICT recognized the suspected outbreak, the hospital re-educated the cleaning staff to more frequently change their gloves and other cleaning tools, particularly during and after the cleaning of restrooms. After re-education, the outbreak clone was not isolated in the hospital, suggesting a possibility that the main site of transmission might be the restrooms, as previously suspected [20]. *H. cinaedi* can present in a coccoid form like *H. pylori* [2], which likely survives some hospital environments for certain periods, potentially facilitating patient-to-patent transmission. As it is also difficult to isolate *H. pylori* from environmental samples, it may be impossible at present to perform systematic WGS analyses of environmental isolates similar to that reported for meticillin-resistant *S. aureus* [47] and*Enterococcus faecium*[27]. However, it may be possible to perform surveillance using culture-independent techniques, such as PCR, in outbreak investigations and this would provide valuable information that complements the WGS analysis of isolates from patients.

The complicated population structure of the *H. cinaedi* strains obtained in this hospital during the study period is also intriguing in terms of the invasion of locally circulating clones into hospitals. The isolate from patient P15, which was initially defined by the ICT as an outbreak strain, appears closely but not directly related to the outbreak clone. This strain appears to represent one of the regionally circulating cluster A clones (Fig. S1). The isolates from P20/P21 also belong to cluster A, but are distinct from the outbreak clone. Thus, it is most likely that multiple invasions of the regionally circulating cluster A clones into the hospital occurred during the study period. Similarly, infections of cluster B isolates, which occurred during the same period and in the same ward (ward Fa), were most likely caused by invasions of multiple cluster B clones into the hospital.

Finally, it should also be mentioned that a subclone with an acquired higher FQ resistance (Fig. 4, Table S4) and the patient (P11) in which this subclone emerged were identified based on the precise genetic relationship between the outbreak isolates revealed by WGS. As the history of FQ administration in patient P11 was also confirmed, this finding represents a clear example of the continuing selection for higher FQ resistance exerted by the wide use of FQs in hospitals.

## Data bibliography

1. Akiyama T, Takeshita N, Ohmagari N *et al.* GenBank/EMBL/DDBJ accession number AP012492 (2012).
2. Kazusa DNA Research Institute, Japan. GenBank/EMBL/DDBJ BioProject ID PRJDB88 (2012).
3. Rimbara E, Mori S, Suzuki M *et al*. GenBank/EMBL/DDBJ accession number AP017374 (2016).
4. Bacteriology II, National Institute of Infectious Diseases, Japan. GenBank/EMBL/DDBJ BioProject ID PRJDB5659 (2017).

## Supplementary Data

Supplementary File 1

Supplementary File 2
