# Supplementary material for: Multi-step genomic dissection of a suspected intra-hospital Helicobacter cinaedi outbreak
Source: Microb Genom. 2019 Jan 17;5(1):e000236. doi: 10.1099/mgen.0.000236 (PMC6412056; doi:10.1099/mgen.0.000236)
Supplement: Supplementary File 1 [file mgen-5-236-s001.pdf]

Multi-step genomic dissection of a suspected intra-hospital *Helicobacter cinaedi* outbreak

Supplementary Date

Supplementary Figures

Fig. S1. The possible strain transmission routes and sources of the 22 *H. cinaedi* strains isolated in Hospital A in Miyazaki.

The 22 *H. cinaedi* strains belong to Clusters A, B, and EX (see Fig. 2 in the main text). The hospitalization periods of each patient (grey bars) and possible strain transmission routes and sources inferred from the genetic relationships of the strains are shown. Pink and blue dotted arrows indicate the transmission of outbreak strains between patients with no hospitalization period overlap, for which the involvement of ACs is inferred (the same as Fig. 4(a) in the main text). Dotted arrows with other colours indicated possible invasions of clones closely related but clearly distinguished by WGS analysis. These clones are most likely circulating in the community in Miyazaki. The time of strain isolation (the number of days after the isolation of P01D0000, the earliest isolate in the present strain set) is shown for each strain at the bottom.

Supplementary Tables

Table S1. Sequencing statuses of the strains used in this study.

Table S2. The numbers of SNPs and informative sites obtained in each step of the phylogenetic analysis.

Table S3. Results of root-to-tip analysis using TempEst.

Table S4. Susceptibilities (MICs; µg/ml) of the 14 outbreak isolates to 9 antimicrobials.

[footnote] MIC determinations were performed only once, except for the type strains. The MICs for the type strain were determined by two independent tests, and when the values in the two tests were different, both values are shown.

Abbreviations and provider companies of each antimicrobial are as follows:

ampicillin (ABPC, Sigma-Aldrich, MO, USA), ceftriaxone (CTRX, Chugai Pharmaceutical, Tokyo, Japan), gentamicin (GM, Wako Pure Chemical Industries, Osaka, Japan), tetracycline (TC, Wako Pure Chemical Industries), minocycline (MINO, Pfizer, NYC, USA), erythromycin (EM,

Sigma-Aldrich), clarithromycin (CAM, Wako Pure Chemical Industries), ciprofloxacin (CPFX, Sigma-Aldrich), and levofloxacin (LVFX, Daiichi Sankyo, Tokyo, Japan).

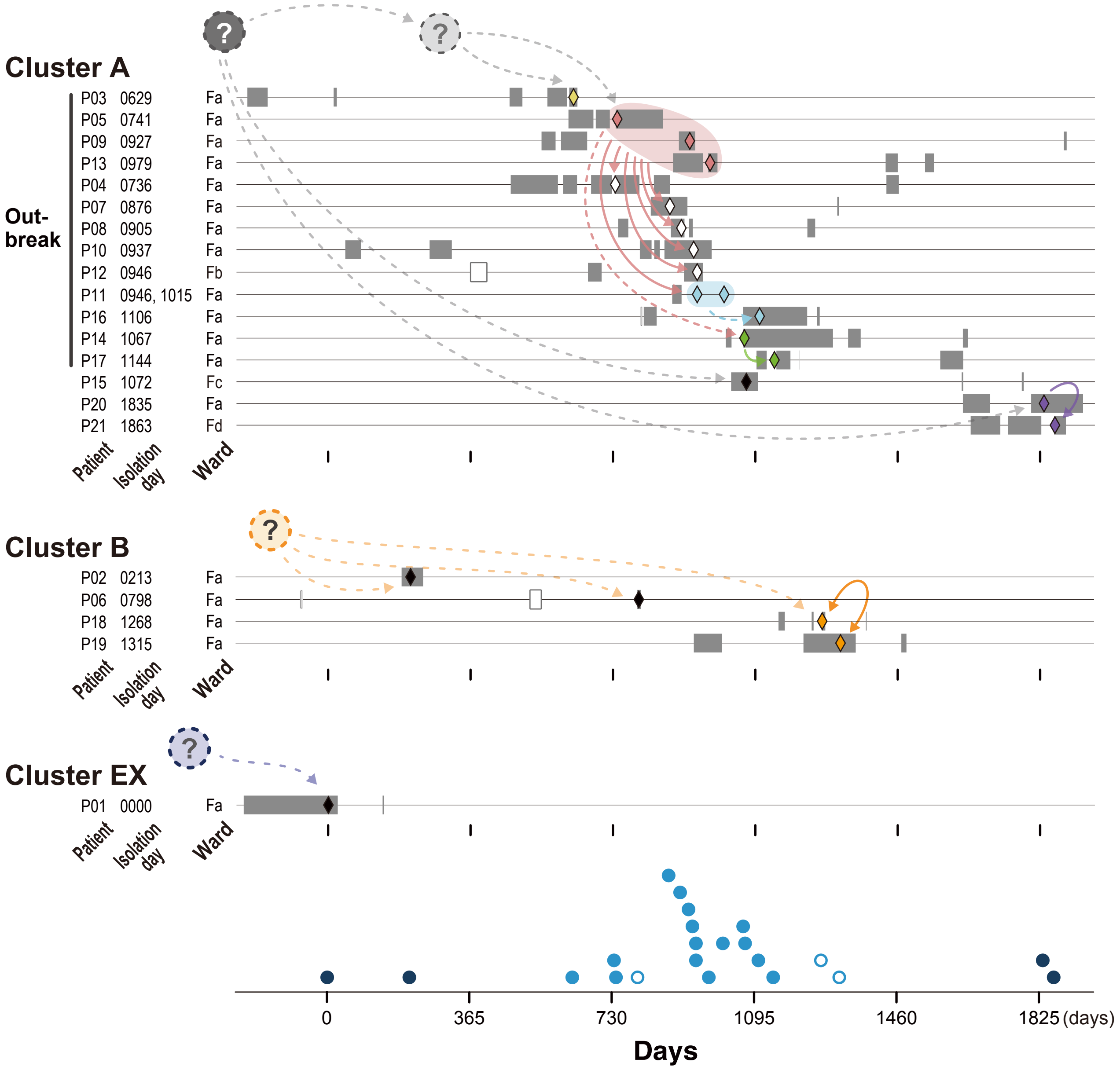
Cluster A
Out-
break
P03 0629 Fa
P05 0741 Fa
P09 0927 Fa
P13 0979 Fa
P04 0736 Fa
P07 0876 Fa
P08 0905 Fa
P10 0937 Fa
P12 0946 Fb
P11 0946, 1015 Fa
P16 1106 Fa
P14 1067 Fa
P17 1144 Fa
P15 1072 Fc
P20 1835 Fa
P21 1863 Fd
Patient
Isolation day
Ward
Cluster B
P02 0213 Fa
P06 0798 Fa
P18 1268 Fa
P19 1315 Fa
Cluster EX
P01 0000 Fa
0
365
730
1095
1460
1825 (days)
Days

Fig. S1.
